# The impact of regional impairment of longitudinal strain and regurgitant jet in aortic regurgitation on myocardial mechanics and postoperative recovery

**DOI:** 10.1186/s44348-024-00042-1

**Published:** 2024-11-26

**Authors:** Ji-won Hwang

**Affiliations:** Division of Cardiology, Department of Medicine, Inje University Ilsan Paik Hospital, Inje University College of Medicine, Goyang, Republic of Korea

Chronic aortic regurgitation (AR) places a significant volume and pressure overload on the left ventricle (LV), resulting in compensatory and damaging changes in the myocardium [1]. The natural progression of AR is well defined, following a gradually progressive clinical course with effects on the LV, including eccentric hypertrophy, fibrosis, and ultimately heart failure, in response to elevated wall stress [2]. LV remodeling and dilatation eventually progress to a stage where the maintenance of wall stress becomes untenable, overwhelming preload reserve. This progression leads to systolic dysfunction, accompanied by a decline in LV compliance and an elevation of LV filling pressures. The increasing wall stress triggers the cardiac fibroblasts to upregulate fibronectin synthesis; the subsequent changes in collagen architecture and myocardial fibrosis further compromise the systolic and diastolic function [3]. A previous cardiac magnetic resonance study reported that chronic AR associated with pressure and volume overload of the LV leads to various forms of myocardial fibrosis, including regional replacement and diffuse interstitial fibrosis [4].

In the 2021 ESC/EACTS guidelines on valvular heart disease, parameters obtained through 2- or 3-dimensional echocardiography speckle-tracking imaging, such as LV global longitudinal strain may be useful, particularly in patients with borderline LV ejection fraction, aiding in the decision-making process for surgical intervention in patients with AR [5]. The evaluation of global longitudinal strain (GLS) using speckle-tracking echocardiography is an increasingly promising technique that has garnered considerable interest. GLS appears to be a more sensitive marker for early detection of LV dysfunction in various valvular diseases, including chronic AR. Even patients with asymptomatic AR exhibit subclinical LV longitudinal axis dysfunction [6, 7]. In asymptomatic patients with greater than moderate chronic AR and preserved LV systolic function, subsequent evaluation using global strain as an alternative staging tool revealed that worsening LV GLS is associated with mortality. The assessment of global strain not only provides incremental prognostic value but also enhances reclassification for improves risk stratification [8, 9].

Literature addressing the association between the impact or role of the regurgitant jet in AR and LV remodeling or mechanics, is rare. However, reports indicates that significant AR influences the regional wall motion of the LV during exercise, impacting the accuracy of exercise echocardiography for coronary artery disease diagnosis [10]. Additionally, a similar finding reported that the degree of wall motion change after exercise in patients with significant AR was not associated with coronary artery disease. Instead, it showed correlation with the severity of volume overload, exercise-induced preload changes, and the direction of the AR jet [11, 12]. These briefly address the role of AR jets in such outcomes.

In this issue of the *Journal of Cardiovascular Imaging*, Joana Ferreira et al. [13] offer valuable insights into the impact of AR on myocardial mechanics, particularly focusing on the regional impairment of longitudinal strain. The authors aimed to investigate whether the regurgitant jet directly influences the myocardial wall, affecting regional longitudinal strain and, subsequently, how this pattern may evolve postoperatively. The study’s implications are significant, introducing the concept of localized myocardial deformation associated with the dynamics of the regurgitant jet. The observed reduction in regional strain may indicate higher regional wall stress, potentially leading to reduced contractility and intriguingly, local fibrosis. The study suggests a link between this regional pattern and postoperative LV recovery, offering a novel perspective on the complexities of AR-induced myocardial remodeling.

Several considerations should be taken into account. The study’s retrospective nature and relatively small sample size may limit the generalizability of the findings. Future research should prioritize validating these findings in larger cohorts, incorporating extended clinical follow-up, and utilizing advanced imaging modalities such as cardiac magnetic resonance imaging, for a more comprehensive assessment of myocardial fibrosis. Investigating the prognostic implications of this regional pattern in chronic AR and its potential role in guiding clinical decision-making holds promise for further exploration.

## Data Availability

Not applicable.

## Abbreviations

AR: Aortic regurgitation
GLS: Global longitudinal strain
LV: Left ventricle
